# Carbon Dioxide Laser Guidelines

**DOI:** 10.4103/0974-2077.58519

**Published:** 2009

**Authors:** DS Krupa Shankar, M Chakravarthi, Rachana Shilpakar

**Affiliations:** *Professor and Head, Department of Dermatology, Manipal Hospital, Bangalore, Karnataka, India*; 1 *Resident, Department of Dermatology, Manipal Hospital, Bangalore, Karnataka, India*

**Keywords:** CO_2_ laser, CO_2_ pixel, dermatological surgery

## Abstract

The carbon dioxide (CO_2_) laser is a versatile tool that has applications in ablative lasing and caters to the needs of routine dermatological practice as well as the aesthetic, cosmetic and rejuvenation segments. This article details the basics of the laser physics as applicable to the CO_2_ laser and offers guidelines for use in many of the above indications.

## INTRODUCTION

The carbon dioxide (CO_2_) laser is the gold standard in ablative lasers. Detailed knowledge of the machines is essential. Over the past decade, advances in laser technology have allowed dermatologists to improve the appearance of scars and wrinkles and to remove benign skin growths using both ablative and nonablative lasers. CO_2_ laser treatment ensures minimal discomfort and rapid recovery, enabling a quick return to daily routine. The CO_2_ laser emits an invisible infrared beam at 10,600 nm, targeting both intracellular and extracellular water. When light energy is absorbed by water-containing tissue, skin vaporization occurs.

## INDICATIONS

### Therapeutic

Actinic and seborrheic keratosis,[1‐5] warts,[6‐9] moles, skin tags, epidermal and dermal nevi,[10‐15] xanthelasma.[16‐19]

Other conditions that have been shown to respond favorably to CO_2_ laser resurfacing include dermatofibroma,[20] rhinophyma,[21‐25] severe cutaneous photodamage (observed in Favre-Racouchot syndrome), sebaceous hyperplasia, syringomas,[1,26‐29] actinic cheilitis,[30‐33] angiofibroma,[34‐36] scar treatment,[37‐39] keloid,[40‐43] skin cancer,[44‐47] neurofibroma,[48‐50] diffuse actinic keratoses, granuloma pyogenicum,[51] and pearly penile papules.[52]

### Aesthetic

Periorbital and perioral wrinkles,[53‐55] facial resurfacing[56‐60] and acne scars,[61‐65] dyschromias including solar lentigines.[66,67]

## CONTRAINDICATIONS

Isotretinoin use within the previous six months, active cutaneous bacterial or viral infection in the area to be treated, history of keloid formation or hypertrophic scarring, ongoing ultraviolet exposure, prior radiation therapy to treatment area, collagen vascular disease, chemical peel and dermabrasion.

## PREOPERATIVE PREPARATION

### Informed consent

Informed consent should be obtained before the procedure according to guidelines.[68] The consent form should specifically state the possible postoperative appearance of the treated area, possible pigmentation changes and need for post-treatment care.

### Position

Position the patient according to the area of lesion such that the area to be treated is close to the laser [Table 1].

**Table 1:** Appropriate positioning of the area to be treated

Area to be treated	Position
Face, chest and abdomen	Supine position
Sides of face, neck and body	Lateral position
Nape of neck and back	Prone position
Palms	Supine position with palms above his head
Soles	Prone position with extended ankle

### Aseptic measures

Gloves, mask and cap should be used by surgeons and assistants. Clean the area with povidone iodine 5% solution (spirit should not be used because it is inflammable).

### Anesthesia

Depending upon the site and type of lesions, one of the following types of anesthesia can be given:

#### Topical anesthesia

Eutectic Mixture of Local Anesthesia (EMLA) cream is used. Apply 2mg/cm^2^ topically under occlusion for 60 min. The occlusion should be removed just before the procedure.

#### Local infiltration

Lignocaine 2% with or without adrenaline 1:100000 is used. Dosage of lignocaine plain is 3 mg/kg and lignocaine with adrenaline is 7 mg/kg. Lignocaine with adrenaline should be avoided at areas with end arteries like fingers, toes, earlobes, nose, and penis. Local anesthesia (LA) is injected as follows:

- Using 30G needle with bevel pointing upward LA is injected immediately below the planned area of laser. Pinching the lesion before injection will reduce the pain.
- In case of palms and soles, insert the needle with 45° angulation to the skin surface.
- Inject the anesthesia while withdrawing and slowly to minimize the pain.
- Insert the needle at a distance from the lesion such that the tip of the needle is below the lesion after it is pushed in to its full length, failing which anesthesia will be deposited distal to the lesion
- Anesthesia must be infiltrated slowly and not pushed in briskly to avoid pain.

#### Ring block

Ring block is employed to anesthetize fingers, toes and penis. The needle is inserted at the base of the fingers and toes on either side or a ring of anesthesia is deposited around the digit. The LA is injected while withdrawing. A distal digital nerve block on either sides of lateral nail folds can supplement a ring block for nail surgeries. In case of penile region, LA is given at the base of the shaft.

#### Field block

LA is infiltrated circumferentially around the site blocking the nerve impulse from leaving the area. The actual surgical site is not injected. They are particularly useful when a large area needs to be anesthetized.

### Eye protection

Patient's eye should be protected with the eye shield or with wet gauze. Dermatologist and assistants should use wavelength-rated spectacles.

## GENERAL INSTRUCTIONS FOR THE OPERATION OF LASER

Hold the hand piece perpendicular to the lesion and press the foot pedal to fire the laser. Vaporize the lesion in coiled, whorled, centrifugal, vertical or horizontal fashion. Vaporize the flat lesions from the top.

Pedunculated lesions can be excised by lasing from the base of the lesion. Hold the lesion with toothed forceps on the top, pull it to the side on the top of the wet gauze (to prevent charring of the normal skin). Always use wet gauze as dry gauze can catch fire.

Wipe the vaporized lesions with wet gauze. Always make sure to dry the area or wipe the water with dry gauze. Look for the raw areas. Coagulate the bleeding spots if any by defocusing the laser beam.

## LASER SPECIFICATIONS FOR VARIOUS DERMATOLOGICAL CONDITIONS AND SPECIAL CONCERNS

In additions to the above general measures that have to be adopted for lasing various cutaneous lesions, there are special considerations for some. The same and the laser settings are summarized in Table 2. Figures 1‐14 show the results after CO_2_ laser in different conditions. It is important to know the relation between the power, irradiance and fluence before performing the procedure [Table 3].

**Figure 1:**
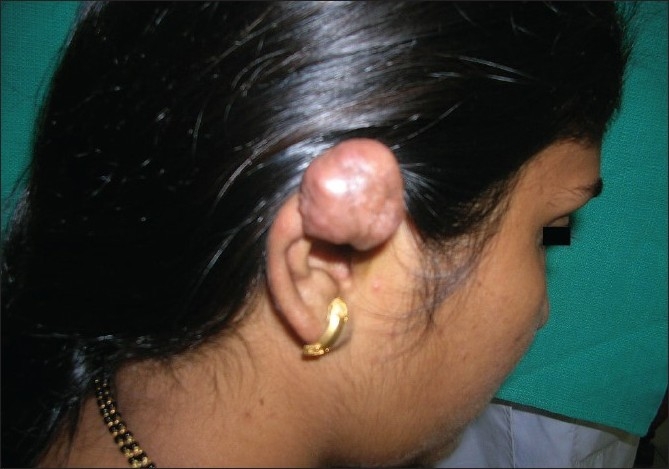
Earlobe keloid before laser

**Figure 2:**
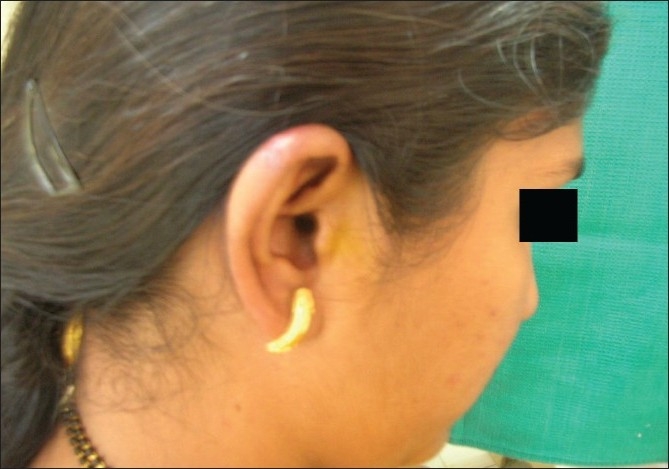
Earlobe keloid after laser

**Figure 3:**
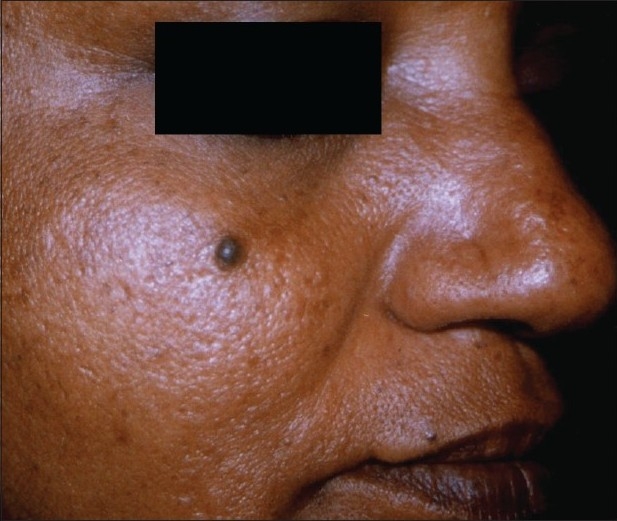
Melanocytic nevi before laser

**Figure 4:**
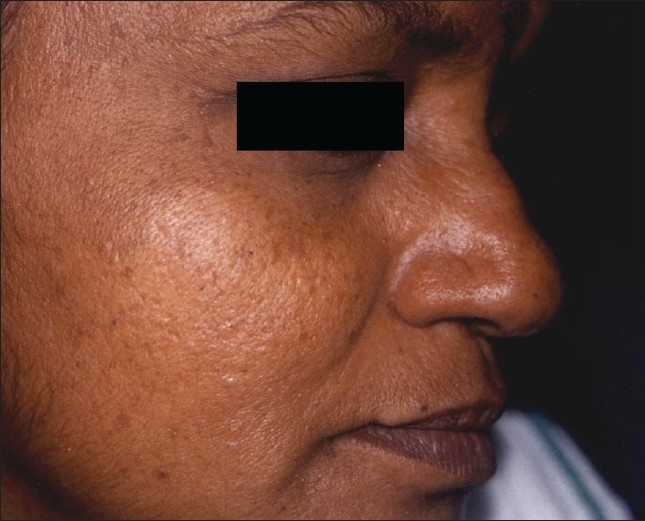
Melanocytic nevi has healed without scarring after laser

**Figure 5:**
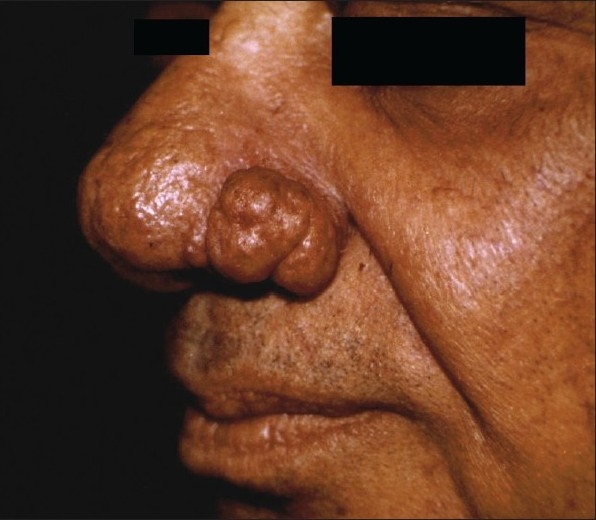
Pre-treatment photograph of rhinophyma

**Figure 6:**
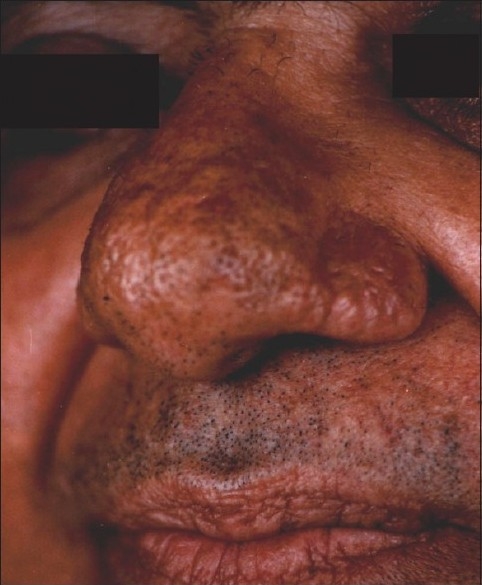
Laser ablation of rhinophyma has healed well with mild residual surface irregularity

**Figure 7:**
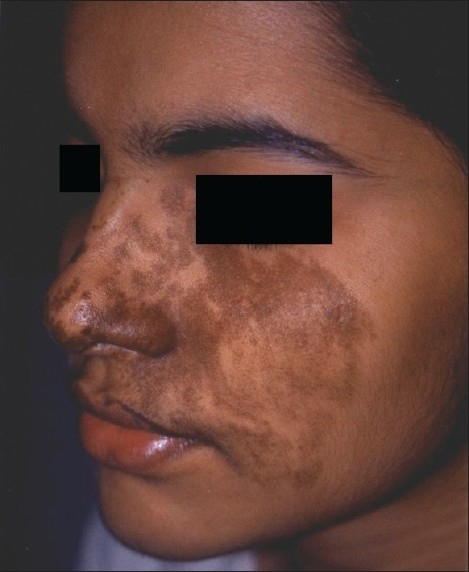
Beckers melanosis on face before treatment

**Figure 8:**
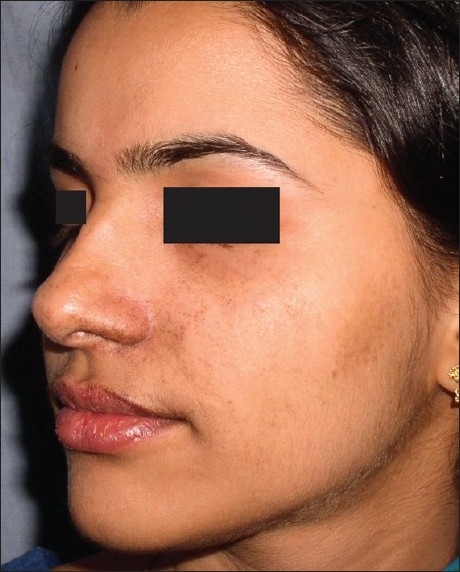
Significant reduction in pigmentation due to Beckers melanosis after laser

**Figure 9:**
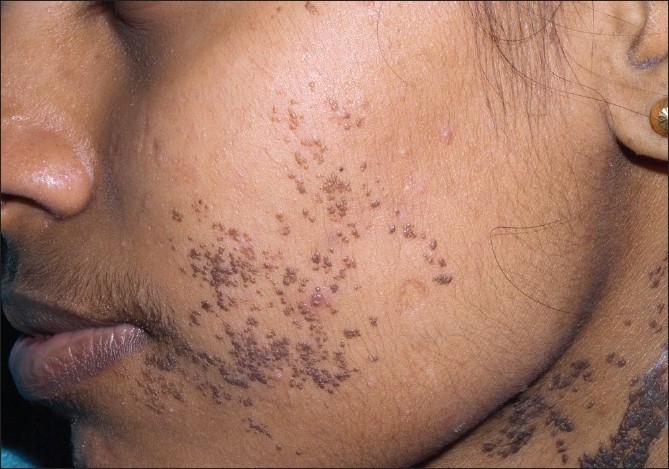
Verrucous epidermal nevus involving left cheek and neck

**Figure 10:**
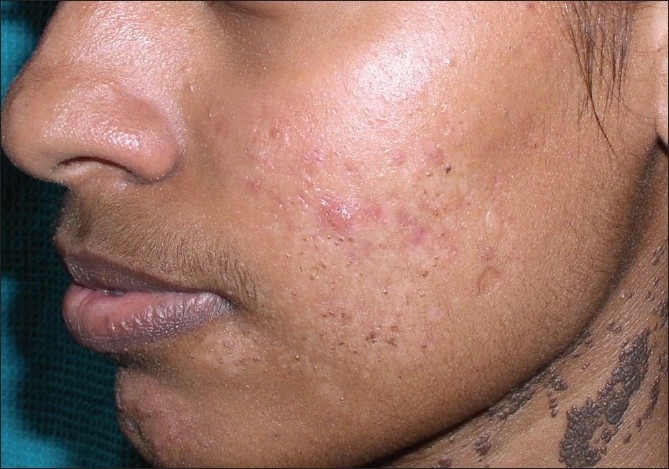
Verrucous epidermal nevus on cheek cleared with mild post-inflammatory hypopigmentation and scarring

**Figure 11:**
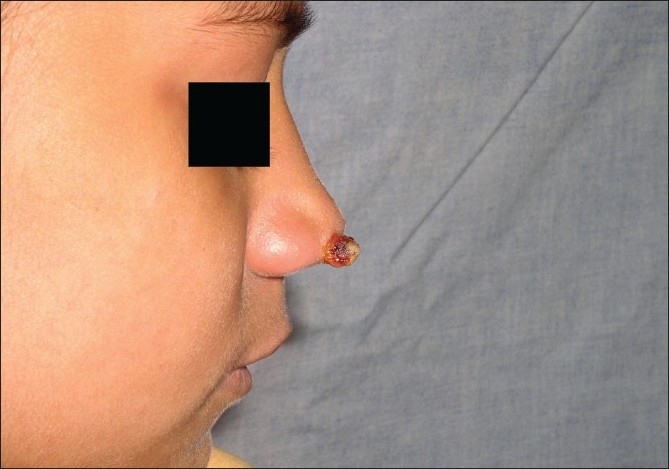
Granuloma telangiectaticum, pre-treatment

**Figure 12:**
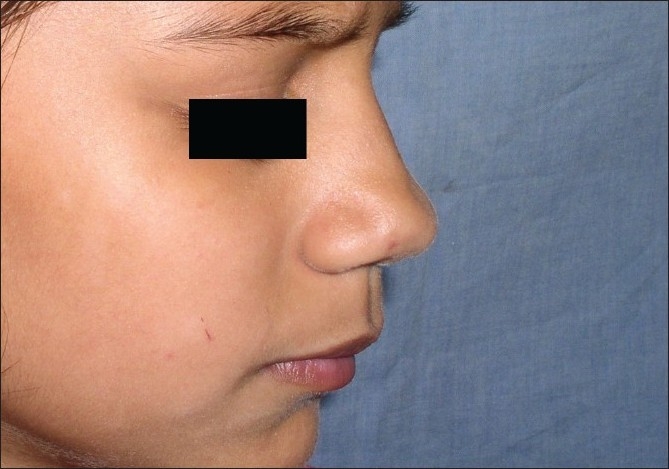
Effective ablation of granuloma telangiectaticum by laser

**Figure 13:**
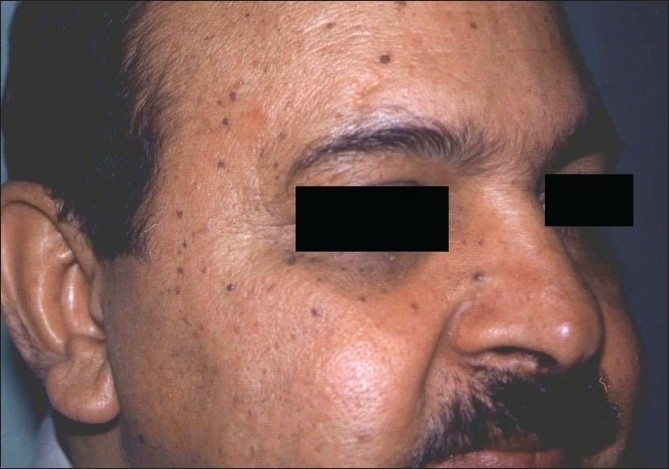
Multiple, brown-black papules of Dermatosis papulosa nigra on face

**Figure 14:**
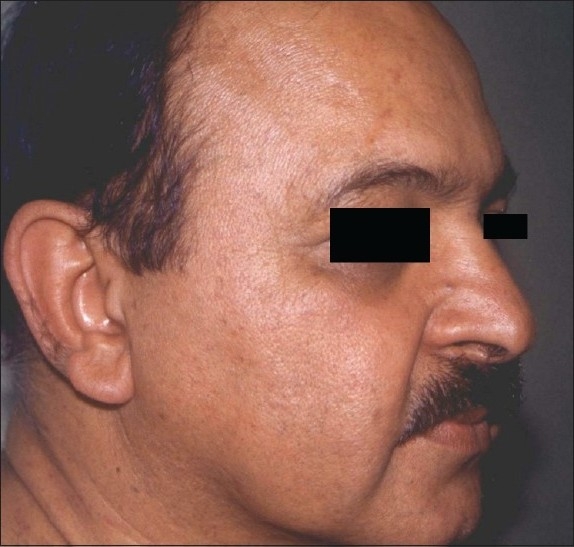
Dermatosis papulosa nigra effectively cleared with laser

**Table 2:** Laser specifications and special considerations for various cutaneous lesions

Dermatological conditions	Laser settings	Comments
Actinic and seborrhoeic keratoses	4 to 7 watts super pulse mode	Topical local anesthesia applied under occlusion at lesions for 45 to 60 min prior to procedure.
Dermatosis papulosa nigra	3.5 to 4.5 watts super-pulsed repeat mode with 0.1 second on and 0.1 second off	Procedure carried out after applying topical LA at each lesion under occlusion.
Warts	9 to 15 watts continuous mode, continuous wave for common warts, use 4 to 6 watts superpulse for flat warts	Precede the vaporization of all types of warts with superficial vaporization of a 1-mm margin of normal skin at half the fluence, before treating the actual lesion, to reduce lesional recurrence.
	Filiform warts can be excised by vaporizing the base	
Palmoplantar warts	8 to 15 watts continuous mode, continuous wave	Precede the vaporization of all types of warts with superficial vaporization of a 1-mm margin of normal skin at half the fluence, before treating the actual lesion, to reduce lesional recurrence
Skin tags	4.5 to 7.5 watts continuous mode	Cut the base of the lesion in focused cutting mode, and avulse the skin tag, in case of giant skin tags, exsanguinate the lesion by applying hemostats to peduncle of anesthetized lesion for 5 min prior to laser avulsion
Epidermal and dermal nevi	4.5 to 7.5 watts super pulse mode	The procedure is repeated till the pigmented areas are visible. Do not go too deep to prevent scar formation
Intradermal and melanocytic nevi on face	4.5 to 7.5 watts super pulse mode	Always send the excised specimen for histopathology and keep a close watch for recurrence for lesions with reported junctional activity. Review the patient on Days 30, 120, 360. If any pigment is noted at treated area, vaporize and repeat follow-up as above
Syringomas, angiofibroma, sebaceous hyperplasia, senile comedones	4.5 to 6.5 watts super pulse mode	In case of syringomas, mark all the lesions with skin marking pen, as they will be rendered invisible after infiltration of anesthetic. The marks must be made with a thin-tipped surgical pen and must circumambulate each lesion
Scars	2.5 to 4.5 watts super pulse mode	
Granuloma pyogenicum	9 to 15 watts continuous mode, continuous wave	Coagulate the lesion including the cuff with slight defocusing to avoid puncturing the lesions which will lead to torrential bleeding
		To attain hemostasis during the procedure, pinch the lesion between the thumb and index finger of the left, hand or apply tourniquet at proximal end
Earlobe keloids	9 to 15 watts continuous mode, continuous wave for large earlobe lesions and 4 to 7 watts super pulse for smaller lesions	Follow up the patient at Day 3, 7, 14 and 30, and inject intralesional triamcinalone at site of healed keloids showing early signs of recurrence
Mucocele	3.5 to 4.5 watts super pulse mode	Mark the outer border of the lesion with dotted lines
Pearly penile papules	3.5 to 4.5 watts super pulse mode using single fixed pulses of 0.1 to 0.5 sec	
Nail bed reconstruction	8 to 12 watts continuous mode	Mark the part of the nail to be avulsed with marker pen. Vaporize the nail in vertical fashion running from the proximal to distal end over the marked line. Separate the nail fold from the nail bed with nail elevator, separate proximal and lateral nail folds from nail plate with curved nail elevator. Avulse the part of the nail from the laser marked line to the lateral nail fold. Vaporize the overhanging mass of lateral nail fold tissue that contributes to onychocryptosis
Nail bed biopsy	6.5 to 8.5 watts super pulse mode	Mark a round of 4 to 5 mm on the nail plate just above the site of biopsy
		Perform the punch biopsy from the nail bed, the size being 1 mm lesser than the avulsed nail plate
		Put back the circular piece of nail bed on the top of the biopsy area to seal the wound

**Table 3:** The relation between irradiance and fluence

Power	Irradiance [w/cm^2^]	Fluence
0.5	6369.43	5.14
1.0	12738.85	11.46
2.0	25477.7	22.93
3.0	38216.56	34.39
4.2	53503.18	48.15
6	76433.12	68.79
6.3	80254.78	72.23
9	114547.54	103.09

## POSTOPERATIVE CARE

- Always apply hydrocolloid dressings on facial procedures, never undertake a facial procedure, if hydrocolloid dressings are unavailable. [See Appendix for instructions on use of hydrocolloid dressings].
- Apply topical antibiotics for the superficial lesions for one week.
- Allow the scabs to fall on own. Avoid picking.
- Emphasize on sunscreen application three times a day from day one for the lesions on the face and neck.
- Treat for post-inflammatory hyperpigmentation if any with Kligman's formula.
- Allow occlusive pressure dressing to remain in place for three to seven days.
- Look for healthy granulation tissue after removal of the occlusive dressing.
- Avoid contact with dust. Use handyplast if needed for a couple of days for protection.

**Appendix:** 

How to use hydrocolloid dressings?
Remove the dressing before bath
Wipe the pus-like material with wet cotton
Wash the area with soap and water when you take bath
Press the area dry after bath
Paint the area and skin around it with povidone iodine 5% solution Wait for 3 min for the solution to dry
Apply the dressing, so that the sticky side of the dressing which adheres to the paper sticks to the wound
Please remember that when you change the dressing you will find a yellowish brown material which may look and smell like pus, but this is not pus, it is the material in the dressing which melts when it comes into contact with the wound
Calibration of CO_2_ laser fluence[69]
Power = joules/sec watts
Spot size = πR^2^
R = Radius = Diameter/2 cm
Irradiance = Power/spot size
Fluence = Irradiance × Time in sec
If
Diameter = 0.1 mm = 0.01 cm
Time = 0.9 m sec = 0.0009 sec
Radius = 0.005 cm
Radius^2^ = 0.000025 cm
Spot size = πR^2^ = 0.00007857
Calibration of CO_2_ pixel laser
W = J/sec
21 W = 21J/1 sec ⇒ 21W × 1 sec = 21J
⇒ 21 W × 0.5 sec = 10.5J
We are using the 7*7 tip, hence 49 pixel dots.
For each pixel dot: 10.5 W/49 pixels = 0.21 W/pixel dot
The diameter of each pixel dot is 100 micron:
J = W/A ⇒ 0.21/(0.1)2*II/4 = 26.75 J/P/cm^2^

## COMPLICATIONS

Minor complications although frequent, are usually of minimal consequence and include post-inflammatory hyperpigmentation, milia formation, perioral dermatitis, acne and/or rosacea exacerbation and contact dermatitis. Hyperpigmentation or erythema over the treated area is common in colored skin and causes anxiety to patients. However, this is temporary, lasting for only about six weeks and gradually improves.

More serious complications include localized viral, bacterial, and candidial infection, delayed hypopigmentation, persistent erythema, and prolonged healing. The most severe complications are hypertrophic scarring, disseminated infection, and ectropion. Early detection of complications and rapid institution of appropriate therapy are extremely important. Delay in treatment can have severe deleterious consequences including permanent scarring and dyspigmentation.

## PRACTICAL TIPS ON USE OF CO_2_ LASER

- Always use hand piece pointer on skin to cut.
- Remember, lens focuses beam and renders it collimated.
- Moving hand piece away [defocusing] leads to logarithmic fall in irradiance; use this to coagulate.
- Super-pulse CO_2_ laser reduces dwell time, maximizes power.
- Use continuous wave in highly vascular lesions and areas, debulking and where esthetics is not an issue *e.g.,* foot.
- Under-treat, eschew therapeutic greed.
- Laser settings in texts are often for collimated hand pieces, read carefully before applying. One-third to one-fourth the irradiance suggested in the texts seems to deliver the results.
- The newer CO_2_ lasers with advanced output control software when used in the super-pulsed mode for carrying out free hand procedures are versatile devices with numerous therapeutic options.

## GUIDELINES FOR CO_2_ PIXEL LASER

- Apply topical anesthesia liberally. Occlude the anesthetic cream with provided plastic sheets and 3M transpore and leave it for 30-45 min.
- After 30-45 min, remove the occlusion and wipe the anesthesia completely with dry gauze.
- Set the pixel laser at 21 watts.
- Give single pass using 7*7 tip, that is, 49 pixel dots. Avoid overlapping but give two passes if scars are deep.
- Apply hydrocolloid dressing for 12 h.
- Procedure has to be repeated every month for four months.

## Multiple-Choice Questions

1. Which of the following is true about CO_2_ laser?
  - It is an ablative laser
  - It is a non-ablative laser
  - It is a semi-ablative laser
  - It is a minimally ablative laser
2. The wavelength of the CO_2_ laser is
  - 10,600 nm
  - 1,064 nm
  - 2,640 mm
  - 10,640 nm
3. The chromophore for CO_2_ laser is
  - Air
  - Water
  - Melanin
  - Hemoglobin
4. The following is not an absolute contraindication for CO_2_ laser therapy:
  - Patient on isotretinoin
  - Keloidal tendency
  - Active viral infection
  - Skin phototype 4 and 5
5. The following must not be used to sterilize the treatment area in CO_2_ laser therapy:
  - Povidone iodine
  - Chlorhexidine
  - Cetrimide
  - Ethanol
6. This equipment is mandatory while carrying out a CO_2_ laser procedure
  - Cold air blower
  - Airconditioning
  - Smoke evacuator
  - Operating theatre lights
7. Dermatosis papulosa nigra is treated with the following type of anesthesia:
  - Ring block
  - Field block
  - Topical anesthesia
  - General anesthesia
8. To cut with the CO_2_ laser, which mode is most suited?
  - Focused
  - Defocused
  - Fractionated
  - Collimated

## Answers

1. a, 2. a, 3. b, 4. d, 5. d, 6. c, 7. c, 8. a.
